# Differential requirement of TIR enzymatic activities in TIR-type immune receptor SNC1-mediated immunity

**DOI:** 10.1093/plphys/kiac452

**Published:** 2022-09-23

**Authors:** Lei Tian, Junxing Lu, Xin Li

**Affiliations:** Michael Smith Laboratories, University of British Columbia, Vancouver, British Columbia V6T 1Z4, Canada; Department of Botany, University of British Columbia, Vancouver, British Columbia V6T 1Z4, Canada; College of Life Science, Chongqing Normal University, Chongqing, 401331, China; Michael Smith Laboratories, University of British Columbia, Vancouver, British Columbia V6T 1Z4, Canada; Department of Botany, University of British Columbia, Vancouver, British Columbia V6T 1Z4, Canada

## Abstract

*Arabidopsis thaliana* TIR-type immune receptor SNC1 (Suppressor of *npr1-1*, constitutive 1) requires NADase, but not the 2′,3′-cAMP/cGMP synthetase activity to trigger *in planta* immune responses.

Dear Editor,

Toll/interleukin-1 receptor (TIR) domain-containing proteins are widespread and play a common role in immunity in most living organisms (Essuman et al., 2018). In land plants, TIR domains can exist in TIR-only proteins, or as parts of the intracellular nucleotide-binding leucine-rich repeat (NLR) immune receptors (TIR-NLRs or TNLs) (Jones et al., 2016; Lapin et al., 2022). Like animal SARM1 (sterile alpha and TIR motif–containing protein 1) and bacterial TIR-containing proteins, cell death responses triggered by plant TIRs/TNLs rely on the oxidized nicotinamide adenine dinucleotide (NAD^+^) hydrolase (NADase) function upon TIR oligomerization (Essuman et al., 2018; Horsefield et al., 2019; Wan et al., 2019). Cryo-EM structural analysis revealed that upon pathogen effector recognition, plant TNLs can tetramerize into a “resistosome,” activating their TIR NADase activity (Ma et al., 2020; Martin et al., 2020). However, the known NADase activity of plant TIR domains is insufficient to induce cell death, suggestive of other TIR functions contributing to immunity (Duxbury et al., 2020). Recently, a structure-function study on the plant TIR-only protein RBA1 (Response to HopBA1) and the truncated TIR domain of flax (*Linum usitatissimum*) TNL L7 revealed an intriguing 2′,3′-cAMP/cGMP synthetase activity upon binding with double-stranded DNA/RNA (dsDNA/RNA). Unlike the TNL tetramer, TIRs alone can assemble with dsDNA/RNA into a superhelix, yielding two noncanonical cyclic nucleotide monophosphates (cNMPs), 2′,3′-cAMP/cGMP. The production of 2′,3′-cAMP/cGMP is indispensable for TIR-mediated cell death in *Nicotiana benthamiana* (Yu et al., 2022). However, it remains unclear whether the full-length canonical TNLs exhibit 2′,3′-cAMP/cGMP synthetase activity *in planta* at organismal levels. Here, we show that the typical Arabidopsis (*Arabidopsis thaliana*) TNL SNC1 (Suppressor of *npr1-1*, constitutive 1) requires NADase activity to trigger plant immune responses, while its 2′,3′-cAMP/cGMP synthetase activity is not needed.

Structure-based mutagenesis analyses of L7 TIR and RBA1 revealed that a few amino acids are essential for the TIR-dsDNA/RNA superhelix formation and the 2′,3′-cAMP/cGMP synthetase activity. Several basic lysine (K) residues from αD helix of L7 TIR are in direct contact with dsDNA/RNA, and mutating them to alanine (A) impaired the superhelix formation and 2′,3′-cAMP/cGMP production. Mutations of the equivalent K residues in RBA1 had similar effects, blocking RBA1-mediated cell death in *N. benthamiana* despite an intact NADase activity. However, when the proposed NADase catalytic glutamic acid (E) was mutated to A, both NADase and 2′,3′-cAMP/cGMP synthetase activities were abolished, indicating the essential roles of this E to both enzyme functions (Yu et al., 2022). Additionally, one conserved cysteine (C) close to the E residue contributed to the 2′,3′-cAMP/cGMP synthetase, but not the NADase activity.

Based on these findings, we tested the requirements of the abovementioned C, E, and K residues to the typical TNL SNC1 through site-directed mutagenesis analysis. The amino acid sequence of SNC1 TIR domain was aligned with the TIRs of L7 and RBA1 to identify the equivalent residues in SNC1 (Supplemental Figure S1). Aside from the conserved C90 and E93, there are three Ks (K129, K136, K138), likely equivalent to the ones in L7 TIR and RBA1 (Supplemental Figure S1). Structural superimposition of the SNC1 TIR with L7 TIR confirmed the similar positions of these Ks in the αD helix region (Supplemental Figure S2). Thus, we introduced C90A, E93A, K129A/K136A/K138A (KKK/AAA hereafter) mutations into SNC1 separately to analyze their effects on SNC1-mediated immunity (Supplemental Table S1, Supplemental Figure S3).

Consistent with previous data (Horsefield et al., 2019; Wan et al., 2019), expression of SNC1 in *N. benthamiana* resulted in a strong cell death response, and cell death was completely abolished by the E93A mutation (Figure 1, A and B). The loss of cell death was not caused by reduced SNC1 protein levels, confirming that NADase activity has no effect on SNC1 protein accumulation (Figure 1C). Surprisingly, the C90A and KKK/AAA mutations, which were predicted to impair the 2′,3′-cAMP/cGMP synthetase activity of the TIR domains, were unable to suppress SNC1-mediated cell death (Figure 1, A and B). Likewise, these mutations did not affect SNC1 protein levels (Figure 1C). These results suggest that the full-length TNL SNC1 may not assemble into a superhelix with dsDNA/RNA as 2′,3′-cAMP/cGMP synthetase during immune activation. As TNL oligomerization is required for the NADase activity and immune responses in plants (Ma et al., 2020; Martin et al., 2020) and SNC1 is known to self-associate (Xu et al., 2014), we then tested if these mutations could affect the self-association of SNC1 *in planta*. As shown in Figure 1D, all SNC1 mutants retained their self-association abilities, consistent with previous observation that TIR NADase activity is downstream of self-association, and the E to A mutation does not affect TIR oligomerization (Wan et al., 2019).

**Figure 1:**
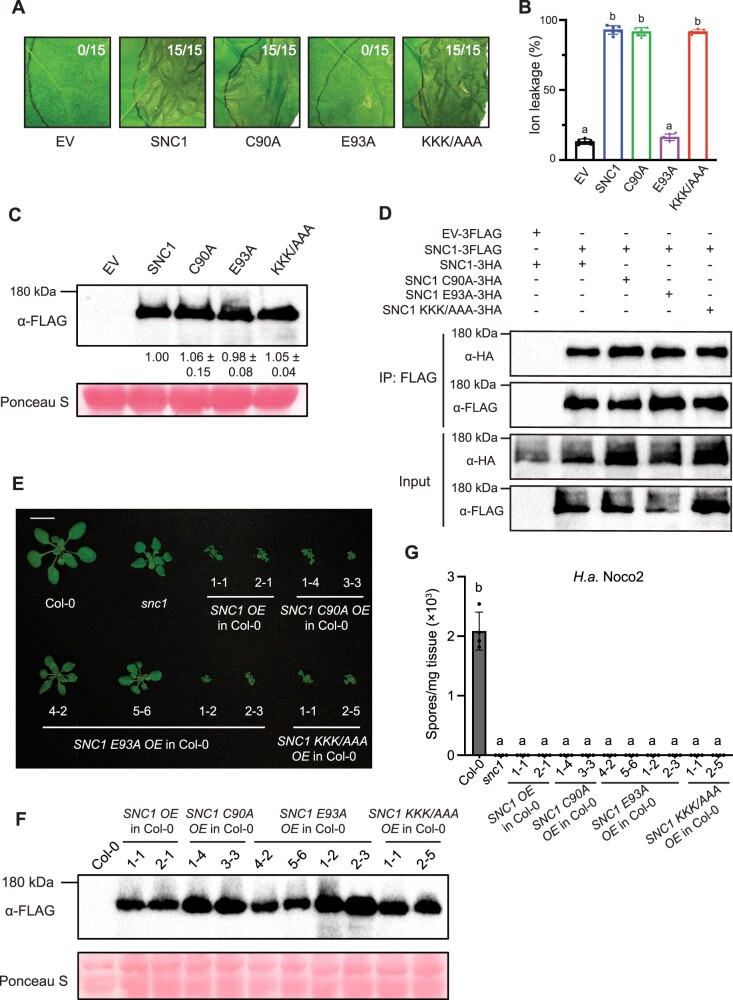
Site-directed mutagenesis analysis of SNC1. A, Cell death analysis in the *N. benthamiana* leaves expressing the control (EV) or the indicated SNC1 proteins. *Nicotiana benthamiana* leaves were infiltrated with *Agrobacterium tumefaciens* expressing EV-3FLAG (EV) or SNC1-3FLAG, SNC1 C90A-3FLAG, SNC1 E93A-3FLAG, SNC1 KKK/AAA-3FLAG at OD_600_ = 0.4. The pictures were taken at 3 days post-inoculation (dpi). The white numbers in each image represent the numbers of leaves displaying cell death out of the total number of leaves infiltrated (biological repeats). Three independent experiments were carried out with similar results. B, Ion leakage of *N. benthamiana* leaves after *A. tumefaciens* infiltration under the same conditions as in (A). Statistical analysis was carried out with one-way ANOVA followed by Tukey’s post hoc test. Statistical signiﬁcance is indicated by different letters (*P* < 0.01). Error bars represent means ± SD (*n* = 6). Three independent experiments were carried out with similar results. C, Immunoblot analysis of protein levels of SNC1-3FLAG, SNC1 C90A-3FLAG, SNC1 E93A-3FLAG, SNC1 KKK/AAA-3FLAG in leaves of (A). Samples were harvested at 30 hours post-inoculation (hpi), when no macroscopic cell death was visible. Equal loading is shown by Ponceau S staining of a nonspecific band. The numbers below represent the normalized ratio between the intensity of the protein band and the Ponceau S band ± SD (*n* = 3). Molecular mass marker in kiloDaltons (kDa) is indicated on the left. D, Immunoprecipitation of SNC1-3HA, SNC1 C90A-3HA, SNC1 E93A-3HA, SNC1 KKK/AAA-3HA by SNC1-3FLAG in *N. benthamiana*. EV-3FLAG served as a negative control. Immunoprecipitation was carried out with anti-FLAG beads. The 3FLAG-tagged proteins were detected using an anti-FLAG antibody. The HA-tagged proteins were detected using an anti-HA antibody. The experiment was repeated three times with similar results. E, Morphology of four-week-old soil-grown plants of Col-0, *snc1*, two independent transgenic lines of *SNC1 OE*, *SNC1 C90A OE*, *SNC1 KKK/AAA OE*, and four independent transgenic lines of *SNC1 E93A OE* into Col-0 background. OE stands for “overexpression” of the indicated *SNC1* allele. Bar = 1 cm. F, Immunoblot analysis of SNC1-3FLAG, SNC1 C90A-3FLAG, SNC1 E93A-3FLAG, SNC1 KKK/AAA-3FLAG protein levels in the indicated 4-week-old soil-grown *A. thaliana* plants. Equal loading is shown by Ponceau S staining of a nonspecific band. G, Quantification of *H.a.* Noco2 sporulation in the indicated genotypes at 7 dpi with 10^5^ spores per ml water. Statistical analysis was carried out with one-way ANOVA followed by Tukey’s post hoc test. Statistical significance is indicated by different letters (*P* < 0.01). Error bars represent means ± SD (*n* = 4). Three independent experiments were carried out with similar results.

In *A. thaliana*, over-accumulation of SNC1 triggers autoimmunity, leading to dwarfism and enhanced resistance to pathogens (Cheng et al., 2011; Xu et al., 2014). Therefore, we overexpressed *SNC1* and its catalytically inactive mutants in wild-type (WT) Col-0 background to analyze the contributions of NADase and 2′,3′-cAMP/cGMP synthetase activities to SNC1-mediated immunity. As expected, overexpression (OE) of *SNC1* rendered plants dwarfed, and the obtained *SNC1 OE* lines were even smaller than the gain-of-function autoimmune mutant *snc1* (Figure 1, E and F; Li et al., 2001; Zhang et al., 2003). Further infection experiments with the oomycete pathogen *Hyaloperonospora arabidopsidis* (*H.a.*) Noco2 confirmed the enhanced immunity in the *SNC1 OE* lines (Figure 1G). Consistent with the *N. benthamiana* cell death results, OE of the putative 2′,3′-cAMP/cGMP synthetase mutants *SNC1 C90A* and *SNC1 KKK/AAA* in WT Col-0 background resulted in transgenic plants as small as the *SNC1 OE* lines (Figure 1, E and F). These *OE* lines were also highly resistant to *H.a.* Noco2 (Figure 1G).

Unexpectedly, we obtained a series of plants with different sizes when overexpressing the catalytically inactive mutant *SNC1 E93A* in WT Col-0 background, and they were comparably resistant to *H.a.* Noco2 (Figure 1, E–G). Interestingly, the plant size of *SNC1 E93A OE* lines reverse associated with the SNC1 E93A protein level (Figure 1F). Given the presence of WT SNC1 in the Col-0 background, we reasoned that the autoimmunity caused by *SNC1 E93A OE* may come from the stabilization of the native SNC1 oligomeric resistosome by SNC1 E93A. To avoid the influence from the endogenous WT SNC1, we then overexpressed all the *SNC1* mutants in the loss-of-function *snc1-r1* background (Zhang et al., 2003), which carries a short deletion in *snc1*. OE of *SNC1*, *SNC1 C90A*, *SNC1 KKK/AAA* in *snc1-r1* background still caused autoimmunity (Figure 2). However, all the *SNC1 E93A OE* lines remained as big as *snc1-r1* regardless of high protein expression (Figure 2, A and B), and they showed similar susceptibility to *H.a.* Noco2 as *snc1-r1* (Figure 2C). Collectively, these data indicate an essential role of TIR NADase, but not the 2′,3′-cAMP/cGMP synthetase, activity in SNC1-mediated autoimmunity in *A. thaliana*.

**Figure 2:**
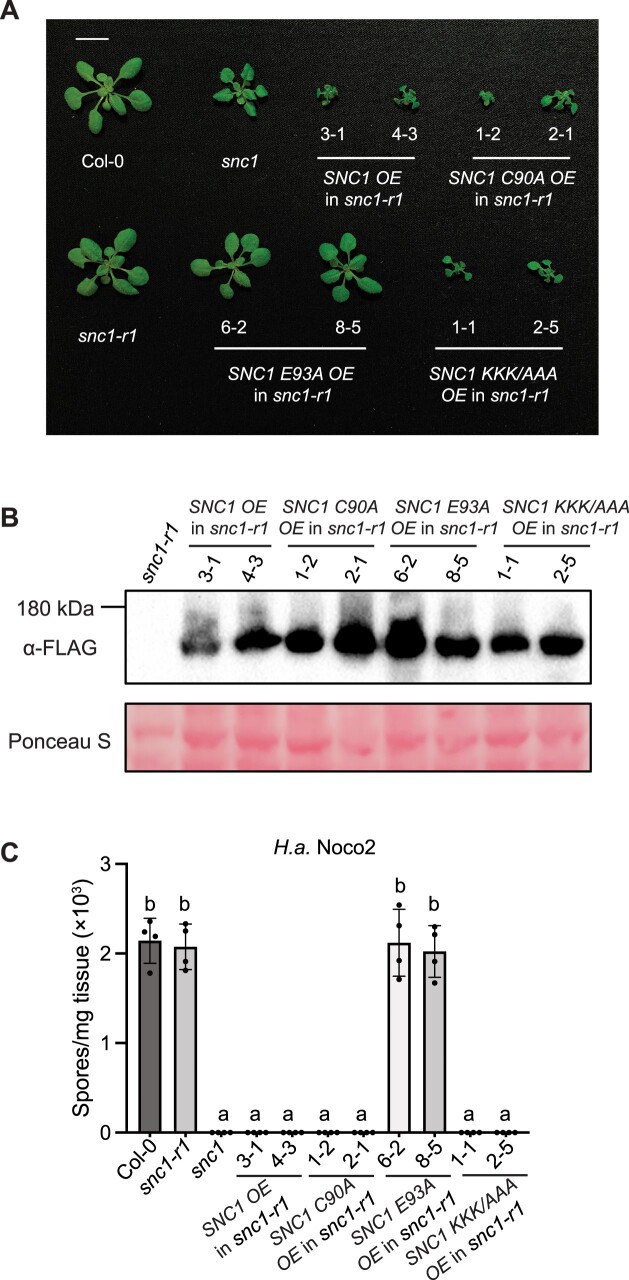
Functional analysis of NADase and 2′,3′-cAMP/cGMP synthetase activities of SNC1 in *A. thaliana* loss-of-function *snc1-r1* background. A, Morphology of 4-week-old soil-grown plants of Col-0, *snc1*, *snc1-r1*, two independent transgenic lines of *SNC1 OE*, *SNC1 C90A OE*, *SNC1 E93A OE*, and *SNC1 KKK/AAA OE* each into the *snc1-r1* background (the same Col-0 and *snc1* plants were used for imaging as in Figure 1E). Bar = 1cm. B, Immunoblot analysis of SNC1-3FLAG, SNC1 C90A-3FLAG, SNC1 E93A-3FLAG, and SNC1 KKK/AAA-3FLAG protein levels in the indicated 4-week-old soil-grown *A. thaliana* plants. Equal loading is shown by Ponceau S staining of a nonspecific band. C, Quantification of *H.a.* Noco2 sporulation in the indicated genotypes at 7 dpi with 10^5^ spores per ml water. Statistical analysis was carried out with one-way ANOVA followed by Tukey’s post hoc test. Statistical significance is indicated by different letters (*P* < 0.01). Error bars represent means ± SD (*n* = 4). Three independent experiments were carried out with similar results.

In summary, our study reveals that the NADase function of TIR is indispensable for both TNL SNC1-mediated immune cell death in *N. benthamiana* and autoimmunity in *A. thaliana*. However, SNC1 does not seem to require a 2′,3′-cAMP/cGMP synthetase activity to activate plant immune responses, although chemical detections of 2′,3′-cAMP/cGMP molecules in these *SNC1*-expressing plants are needed for further support. Our study raises cautions about the biological importance of the TIR 2′,3′-cAMP/cGMP synthetase activity in full-length TNLs. 2′,3′-cAMP/cGMP may contribute to TIR-mediated boosting of immunity from the early responses to pathogen-associated molecular patterns (PAMPs) or as an immune-amplifying signal (Tian et al., 2021), which could be masked in our SNC1 OE experiments. Strikingly, two recent reports just uncovered two different ADP derivatives produced by TIR NADase activities of plant TNLs. These molecules may be perceived by two distinct downstream receptors (Huang et al., 2022; Jia et al., 2022). The relationship between 2′,3′-cAMP/cGMP and these ADP derivatives remains unclear. Future investigations on the complete spectrum of *in planta* signaling molecules produced by TNL holoenzymes will help understand the full TNL enzyme activities and the connection of TNL oligomerization to the downstream immune activation in plants.

## Supplemental data

The following materials are available in the online version of this article.

**Supplemental Figure S1.** Sequence alignment of TIR domains of L7, RBA1, and SNC1.

**Supplemental Figure S2.** Structural superimposition of the TIR domains of SNC1 and L7.

**Supplemental Figure S3.** Generation of catalytically inactive SNC1 mutants.

**Supplemental Table S1.** The list of primers used in this study.

## Funding

This work was supported by funds to X.L. from Canada Foundation for innovation-John R. Evans Leaders Fund (CFI-JELF), the Natural Sciences and Engineering Research Council of Canada (NSERC) Discovery and NSERC-CREATE PRoTECT programs. L.T. and J.L. are partly supported by China Scholarship Council (CSC) scholarships.

*Conflict of interest statement*. None declared.

## Supplementary Material

kiac452_Supplementary_Data

## Notes

L.T. contributed to conceptualization, data curation, validation, investigation, methodology, writing—original draft, project administration; J.L. contributed to validation, methodology; X.L. contributed to conceptualization, data curation, formal analysis, supervision, funding acquisition, project administration, writing—revisions. All authors reviewed the manuscript.

The author responsible for distribution of materials integral to the findings presented in this article in accordance with the policy described in the Instructions for Authors (https://academic.oup.com/plphys/pages/general-instructions) is: Xin Li (xinli@msl.ubc.ca).
