# Supplementary material for: Differential requirement of TIR enzymatic activities in TIR-type immune receptor SNC1-mediated immunity
Source: Plant Physiol. 2022 Sep 23;190(4):2094–8. doi: 10.1093/plphys/kiac452 (PMC9706416; doi:10.1093/plphys/kiac452)
Supplement: kiac452_Supplementary_Data [file kiac452_supplementary_data.pdf]

Differential requirement of TIR enzymatic activities in TNL SNC1-mediated immunity

Supplemental Methods

Construction of plasmids

Overexpression constructs of *SNC1*, *SNC1 C90A*, *SNC1 E93A* and *SNC1 K129A/K136A/K138A* (*SNC1 KKK/AAA*) were made in *pBASTA-3FLAG* and *pCambia1300-3HA* vectors under the 35S promoter. The *SNC1* genes were cloned from *A. thaliana* Col-0 genomic DNA. Overlapping PCR-mediated site-directed mutagenesis method was used to generate the three SNC1 mutants. The fragments were amplified from wild-type *SNC1* template with primers that contain AvrII site and the introduced mutations. All primers used in the study are listed in Table S1. All the *SNC1* mutant constructs were confirmed by Sanger sequencing (Macrogen).

Plant materials and growth conditions

A. thaliana and *N. benthamiana* plants were grown at 22 ºC under a long day condition of 16-hr light/8-hr dark regime.

A. thaliana transformation

The binary constructs were introduced into *Agrobacterium tumefaciens* GV3101 by electroporation and subsequently transformed into *A. thaliana* Col-0 or *snc1-r1* background by the floral-dipping method (Clough & Bent, 1998). Transformants were either selected on soil by spraying the herbicide BASTA (Glufosinate ammonium) or on ½ MS plates with Hygromycin B. Co-segregation analysis was then performed with the transformants in T_2 generations to make sure that the observed phenotypes were due to the transgene overexpression.

Pathogen infection assay

Pathogen infection assays were carried out as described previously (Li et al., 2001). In brief, two-week-old soil-grown seedlings were sprayed with *H.a.* Noco2 conidia spores at a concentration of 10^5 spores per ml water. The plants were then transferred to a humid chamber at

18 °C with 12-hr light/12-hr dark. After 7 days, pathogen sporulation was quantified using a hemocytometer.

Ion leakage measurement in *N. benthamiana*

After agrobacteria infiltration, leaf discs from *N. benthamiana* leaves were collected and washed in 10 ml of ddH_2O overnight at room temperature. Ion leakage was measured with a conductometer (VWR E C METER Model 2052). All samples were then autoclaved before measuring the total ion leakage.

Protein extraction, immunoprecipitation, and western blot analysis

0.1 g soil-grown *A. thaliana* or *N. benthamiana* leaves were collected and extracted by extraction buffer (100 mM Tris-HCl pH 8.0, 0.2% SDS and 2% β-mercaptoethanol). Loading buffer was added to each protein sample and boiled for 8 min, followed by western blot analysis. Protein abundance was quantified using ImageJ (https://imagej.nih.gov/ij/).

Co-immunoprecipitation assay was performed as previously described (Wu et al., 2022). In brief, about 2.0 g *N. benthamiana* leaves expressing the indicated proteins were harvested at 36 hpi and ground into fine powder with liquid nitrogen. Extraction buffer contains 25 mM Tris-HCl pH 7.5, 300 mM NaCl, 5 mM $MgCl_2$, 0.15% Nonidet P-40, 10% Glycerol, 1 mM PMSF, 1× Protease Inhibitor Cocktail (Roche; Cat. #11873580001), and 10 mM DTT. The FLAG-tagged proteins were immunoprecipitated using 20 µl M2 beads (Sigma Cat. #A2220). The anti-HA antibody was from Roche (Cat. #11867423001). The anti-FLAG antibody was from Sigma (Cat. #F1804). Protein abundance was quantified using ImageJ (https://imagej.nih.gov/ij/).

Statistical analysis

Statistical analysis was carried out with one-way ANOVA followed by Tukey's post hoc test. The Scheffé multiple comparison was applied for testing correction. Normality test for all data was done in SPSS. Statistical significance was indicated with different letters. *p* values and sample numbers (n) were elaborated in figure legends.

References

Clough, S. J., & Bent, A. F. (1998). Floral dip: a simplified method for Agrobacterium-mediated transformation of *Arabidopsis thaliana*. *The plant journal*, *16*(6), 735-743.

Li, X., Clarke, J. D., Zhang, Y., & Dong, X. (2001). Activation of an EDS1-mediated R-gene pathway in the snc1 mutant leads to constitutive, NPR1-independent pathogen resistance. *Molecular plant-microbe interactions*, *14*(10), 1131-1139.

Wu, Z., Tian, L., Liu, X., Huang, W., Zhang, Y., & Li, X. (2022). The N-terminally truncated helper NLR NRG1C antagonizes immunity mediated by its full-length neighbors NRG1A and NRG1B. *The Plant Cell*, *34*(5), 1621-1640.

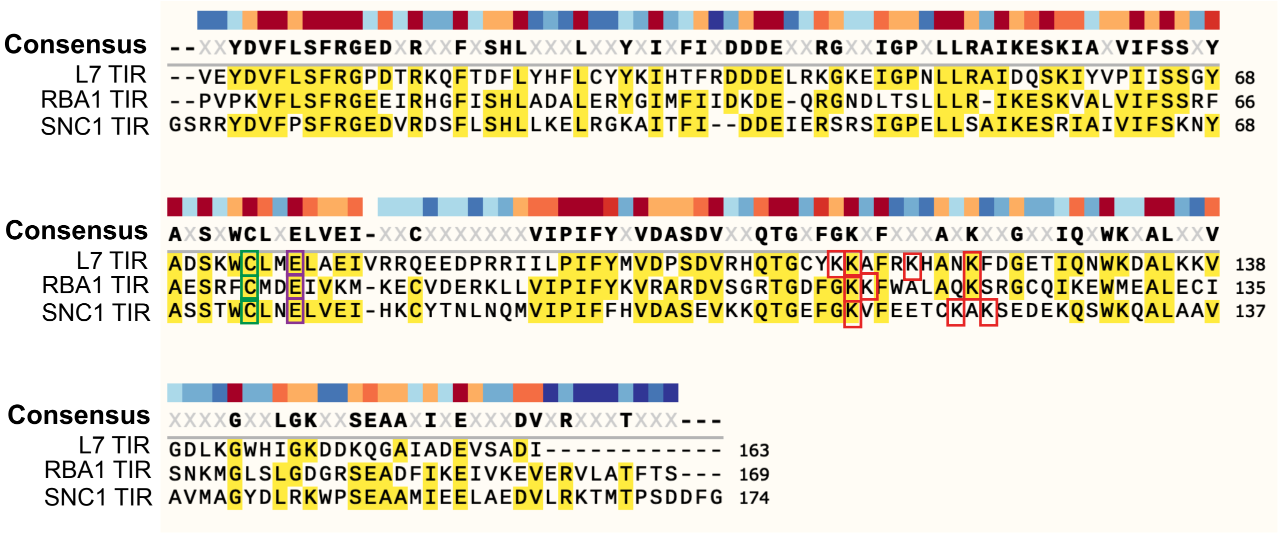

Supplemental Figure S1: Sequence alignment of TIR domains of L7, RBA1 and SNC1.

RBA1 and SNC1 protein sequences were obtained from TAIR, L7 protein sequence was obtained from Genbank. The conserved cysteine (C) and glutamic acid (E) residues are marked with green and purple boxes respectively. Equivalent lysine (K) residues in the αD region are marked with red boxes.

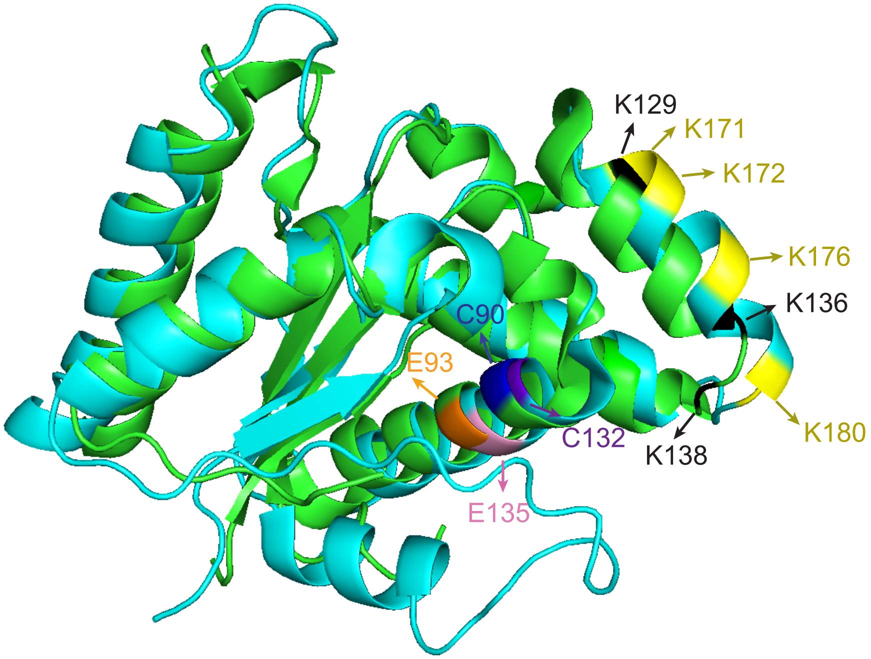

Supplemental Figure S2. Structural superimposition of the TIR domains of SNC1 and L7.

SNC1 TIR (PDB ID: 5TEC) and L7 TIR (PDB ID: 7VU8) are shown in green and cyan respectively. TIR domains were aligned in PyMOL (Schrödinger LLC) using the “super” structural imposition function. The critical amino acid residues are pointed out with arrows in different colors.

Figure S3

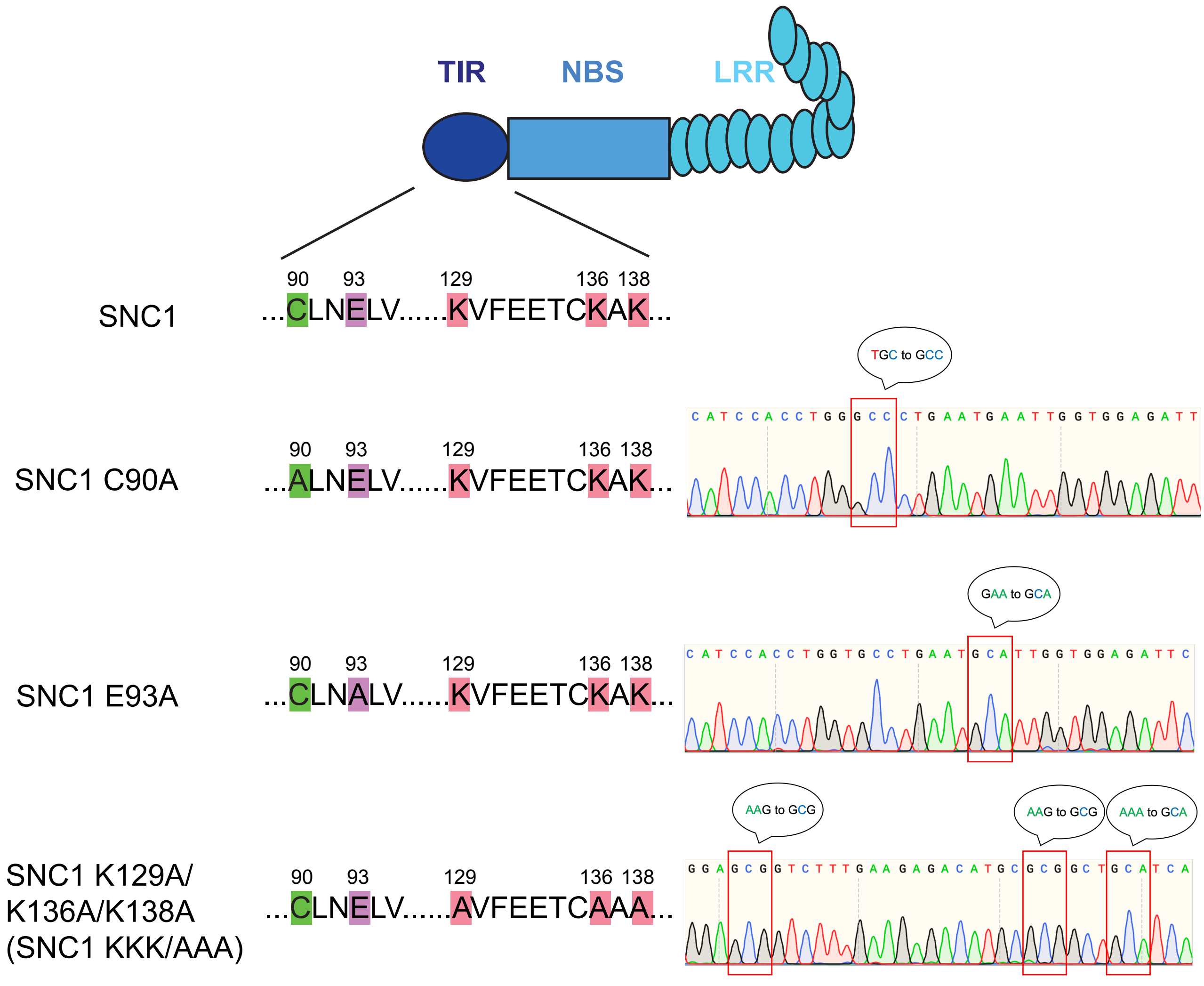

Supplemental Figure S3. Generation of catalytically inactive SNC1 mutants.

SNC1 C90A was made by replacement of the corresponding nucleotides ‘TGC’ with ‘GCC’. SNC1 E93A was made by replacement of the corresponding nucleotides ‘GAA’ with ‘GCA’. SNC1 K129A/K136A/K138A was made by replacement of the corresponding nucleotides ‘AAG’, ‘AAG’, ‘AAA’ with ‘GCG’, ‘GCG’, ‘GCA’. Sanger sequencing results are shown on the right. TIR: Toll/interleukin-1 receptor domain; NBS: nucleotide-binding site; LRR: leucine-rich repeat.

Supplemental Table S1. The list of primers used in this study

Name	Sequence	Purpose
SNC1-KpnI-F	CGGGGTACCATGATGGATACATCCAA	To generate pBASTA SNC1-3FLAG and pCambia1300 SNC1-3HA
SNC1-SpeI(ns)-R	GGACTAGTGTTACCAGAAACAGGAAA	
SNC1-KpnI-F	CGGGGTACCATGATGGATACATCCAA	To generate pBASTA SNC1 C90A-3FLAG and pCambia1300 SNC1 C90A-3HA
SNC1-C90A-R	CCAATTCATTCAGGGCCCAGGTGGATGA	
SNC1-C90A-F	TCATCCACCTGGGCCCTGAATGAATTGG	
SNC1-AvrII-R	TTCGGAGCCTAGGCATCATCTCCATC	
SNC1-KpnI-F	CGGGGTACCATGATGGATACATCCAA	To generate pBASTA SNC1 E93A-3FLAG and pCambia1300 SNC1 E93A-3HA
SNC1-E93A-R	TGAATCTCCACCAATGCATTCAGGCACCA	
SNC1-E93A-F	TGGTGCCTGAATGCATTGGTGGAGATTCA	
SNC1-AvrII-R	TTCGGAGCCTAGGCATCATCTCCATC	
SNC1-KpnI-F	CGGGGTACCATGATGGATACATCCAA	To generate pBASTA SNC1 KKK/AAA-3FLAG and pCambia1300 SNC1 KKK/AAA-3HA
SNC1-K129A-R	GCATGTCTCTTCAAAGACCGCTCCAAATTCGCCG	
SNC1-K136/138A-F	GGTCTTTGAAGAGACATGCGCGGCTGCATCAGAGGATGAGA	
SNC1-AvrII-R	TTCGGAGCCTAGGCATCATCTCCATC	
